# Household Air Pollution and Under-Five Mortality in Bangladesh (2004–2011)

**DOI:** 10.3390/ijerph121012847

**Published:** 2015-10-15

**Authors:** Sabrina Naz, Andrew Page, Kingsley Emwinyore Agho

**Affiliations:** 1 Centre for Health Research, School of Medicine, Western Sydney University, Campbelltown Campus, Locked Bag 1797, Penrith, NSW 2571, Australia; E-Mail: a.page@westernsydney.edu.au; 2 School of Science and Health, Western Sydney University, Campbelltown Campus, Locked Bag 1797, Penrith, NSW 2571, Australia; E-Mail: k.agho@westernsydney.edu.au

**Keywords:** Household air pollution, indoor air pollution, under-five mortality, cooking fuels, Bangladesh

## Abstract

Household air pollution (HAP) is one of the leading causes of respiratory illness and deaths among children under five years in Bangladesh. This study investigates the association between HAP from cooking fuel and under-five mortality using Bangladesh Demographic and Health Survey (BDHS) datasets over the period 2004–2011 (*n* = 18,308 children), and the extent to which this association differed by environmental and behavioral factors affecting level of exposure. The association between HAP and neonatal (age between 0–28 days), infant (age between 0 and 11 months) and under–five (age between 0 and 59 months) mortality was examined using multilevel logistic regression models. HAP was not strongly associated with overall neonatal (OR = 1.49, 95% CI = 1.01–2.22, *p* = 0.043), infant (OR = 1.27, 95% CI = 0.91–1.77, *p* = 0.157) or under-five mortality (OR = 1.14, 95% CI = 0.83–1.55, *p* = 0.422) in the context of overall decreasing trends in under-five mortality. The association was stronger for households with an indoor kitchen using polluting fuels, and in women who had never breastfed. Reductions in exposure to pollution from cooking fuel, given it is a ubiquitous and modifiable risk factor, can result in further declines in under-five mortality with household design and behavioural interventions.

## 1. Introduction

Household air pollution (HAP) from cooking fuel is a substantial cause of respiratory illness and death and remains a major public health concern in the developing world [1,2]. Globally, more than three billion people depend on solid fuels (wood, animal dung, crop residues, charcoal and coal) for cooking and heating and, in the case of rural populations, approximately 90% households use biomass fuels as their primary source of domestic energy [3,4,5]. Polluted indoor air is associated with a range of health damaging pollutants such as fine particles, carbon monoxide (CO_2_), nitrogen oxides (NO_2_), sulphur dioxide (SO_2_), benzene, butadiene, formaldehyde, polyaromatic hydro-carbons and a number of other chemicals [6,7].

The poorest populations of the world are most vulnerable to the effects of household air pollution (HAP), with approximately 4.3 million deaths worldwide has been attributable to HAP in 2012 [8]. HAP has a disproportionately adverse effect on the health of women and children under five years of age, particularly acute respiratory infections (ARI) [4,9] as women are predominantly responsible for cooking in developing societies which usually occurs indoors with fuel burnt in open, poor functioning stoves [4,10]. In addition, children are more vulnerable to air pollution than adults because of their higher oxygen consumption rate for which they inhale more pollutants and also for the fact that their airways are narrower which results more irritation for greater airway obstruction [11,12]. However, young children are at greater risk of exposure from cooking fuel than older children as they spend more time indoors [2,10,13].

Bangladesh is a developing and overpopulated low land country located in South Asia. Nearly, 31% of people in peri-urban and rural areas live in poverty [14]. Approximately 86% of the population still rely primarily on solid fuels as a domestic source of energy, particularly in rural households [14]. Around 3.6% of the total burden of disease in the country has been attributable to HAP and 21% of deaths among children <5 years are associated with ARI [15,16]. Over the past decades, under-five mortality in Bangladesh has dropped by 72% from 144 deaths per 1,000 live births in 1990 to 41 in 2012 [17]. Despite this, pneumonia is still the leading single cause of under-five deaths in Bangladesh, accounting for one-fifth of all deaths [14].

Previous studies in Bangladesh have examined the effects of HAP on respiratory diseases among young children, but have been limited to surveys of limited geographic areas, or hospital based data sources for specific regional populations [18,19,20,21,22,23]. To date, no study for Bangladesh has examined the effect of HAP on nationally representative under-five mortality, and no studies have examined changes over time or investigated the role of environmental and behavioral factors that might affect the level of exposure to HAP (for example, place of residence, breastfeeding status and location of kitchen). Accordingly, the objective of this study was to investigate trends in the association between HAP and under-five mortality, and assess how this is affected by key environmental and behavioral factors using nationally representative data over the period 2004–2011.

## 2. Methods

### 2.1. Data Sources

The data in this study were extracted from the most recent Bangladesh Demographic and Health Survey (BDHS) datasets for the years 2004, 2007 and 2011. The BDHS are nationally representative household surveys conducted every three to four years since 1993 under the authority of the National Institute of Population Research and Training (NIPORT) of the Ministry of Health and Family Welfare, implemented by Mitra and Associates in Dhaka (Bangladesh) with technical assistance from ICF international of Calverton, Maryland, USA, as a part of its Demographic and Health Surveys Program [14,24,25]. The BDHS data were collected by interviewing ever-married women (aged 10–49 years) and men (aged 15–54 years) using a stratified sample of households based on a two-stage cluster design [14,24,25]. A total of 40,439 ever-married women of reproductive age (14,320 from urban and 26,119 from rural areas) were included in the three datasets with a response rate of 98.3% in women across the three datasets (2004, 2007 and 2011). This study was based on information relating to 18,308 singleton live-born children, of whom 923 died in the 5-years prior to the survey. An index period of five years was to minimize recall bias of child birth and death information self-reported by the mother. (Descriptive characteristics of participating women are provided in a Table S1).

#### 2.1.1. Under-Five Mortality

The analysis was carried out for three age groups: neonatal, infant and under-five mortality, using the following definitions:

(1) *Neonatal mortality:* The number of deaths during the first 28 days of life (0–28 days). Defined as, Number of neonatal deaths/Total number of live births

(2) *Infant mortality:* The number of deaths during the first year of life (0–11 months). Defined as, Number of infant deaths/Total number of live births

(3) *Under-five mortality:* The number of deaths before the fifth birthday (0–59 months). Defined as, Number of under-five deaths/Total number of live births

The outcome variables were considered dichotomous for the analysis, where “age at death” was either yes (=1), denoting death occurred during any of these three periods of age, or no (=0), denoting the child survived during the age-period. 

#### 2.1.2. Type of Cooking Fuel

The main exposure variable was type of cooking fuel used in the household. The respondents were asked, “What type of fuel does your household mainly use for cooking?” and in response 12 types of cooking fuel were reported. In the analysis, these fuels were grouped into two categories on the basis of exposure to cooking fuel: “clean fuels” (electricity, liquid petroleum gas, natural gas, and biogas) and “polluting fuels” (kerosene, coal/lignite, charcoal, wood, straw/shrubs/grass, agricultural crops, and animal dung). BDHS analyses have previously classified cooking fuel as “solid” and “non-solid” fuels, where kerosene was categorized in the non-exposed (*i.e.* “clean fuel”) group [14,24,25]. However, many previous studies have reported kerosene as a polluting fuel and have found significant associations between under-five mortality and respiratory illness among children and kerosene fuel use [26,27,28]. For this reason, kerosene was categorized in the polluting fuels group.

#### 2.1.3. Potential Confounders

A household wealth index (categorized as “high income”, “middle income” or “low income”), mother’s education (categorized as “secondary or higher”, “primary” or “no education”) and mother’s working status (“working” or “not working”) were included as markers of socio-economic status, and have previously been identified as potential confounders [27,28,29,30,31,32,33]. The household wealth index was constructed using principal components analysis, with weights for the wealth index calculated by giving scores to the asset variables such as ownership of transport, durable goods and facilities in the household [14,24,25,34]. “Low income” referred to the bottom 40% of households, “middle income” referred to the middle 40% of households, and “high income” referred to the top 20% of households, based on the approach described by Filmer and Pritchett [34]. Mother’s age (age < 20, 20–29, 30–39 or 40–49 years) and wall material of household (“cement/brick” or “non-cement/non-brick”) were also considered as potential confounders of the association between HAP and under-five mortality.

Place of residence (categorized as “urban” or “rural”), breastfeeding status (categorized as ever breastfed “yes” or “no”) of children and location of kitchen (categorized as “separate building/outdoors” or “in the house”) were also considered *a priori* factors that may indicate different levels of exposure to polluting fuels. While not necessarily a modifiable risk factor, place of residence is likely to play an important role in child survival as rural children have a higher risk of death from use of polluting fuels than urban counterparts [27,29,31,32,35,36], and an inside kitchen as an indicator of proximity to polluting fuel use has also been shown to be an important factor associated with greater exposure to HAP [18,20,21,22,29,32,37]. Additionally, breastfeeding has been shown to be a protective factor for under-five mortality, generally in infants [28,29,38,39,40,41], which may attenuate the higher risk associated with HAP exposure. Hence, analyses sought to determine whether the magnitude of the association between HAP and under-five mortality differed by past breastfeeding status.

### 2.2. Statistical Analysis

The association between type of cooking fuels and under-five mortality was investigated using a series of multilevel logistic regression models adjusted for the potential confounders of household wealth, place of residence, mother’s age, mother’s education, mother’s working status, breastfeeding status and wall material of house. Changes in neonatal, infant and under-five mortality rates from HAP over time were also investigated using a trend analysis across 2004, 2007 and 2011 BDHS data by specifying “period” as a continuous variable. To identify the overall effect of HAP from cooking fuels with neonatal, infant and under-five mortality, pooled analyses were also conducted. The extent of divergence or convergence between the slopes of period specific trends within each variable over the study period (2004–2011) was assessed by testing the interaction between period and a given confounding variable using likelihood ratio tests.

Stratified analyses were also conducted by urban and rural areas, breastfeeding status and by location of kitchen (for 2007–2011 only, where information on location of kitchen was available) to determine whether the magnitude of the effect of the exposure on outcomes differed across levels of these variables. Geographical region (“urban” or “rural”), breastfeeding status (ever breastfed “ yes” or “no”) and location of kitchen (“separate building/outdoors” or “in the house”) were each also combined with type of cooking fuel as composite ordinal variables to investigate different level of exposure to HAP for under-five mortality outcomes.

The “Svy” command was used for calculating weighted cumulative incidence estimates of mortality to adjust for the cluster sampling survey design. Multilevel logistic regression models were conducted by using the “xtlogit” command and for likelihood ratio test for interaction “lrtest” command was used. All analyses were carried out in STATA version 13.1 (Stata Corp: College Station, TX, USA).

### 2.3. Ethics

The DHS project sought and obtained the required ethical approvals from ethics committees in Bangladesh before the surveys were conducted. Informed consent was obtained from study participants before they were allowed to participate in the surveys. The survey data sets used in this study were completely anonymous with regard to participate identity.

## 3. Results

**Figure 1:**
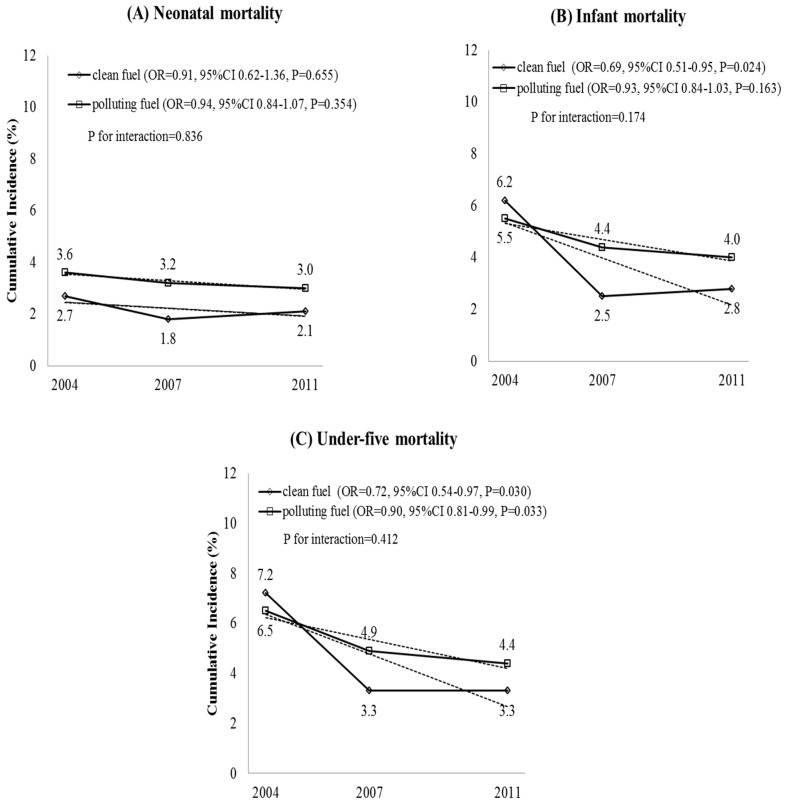
Under-five mortality trend with exposure to cooking fuel in Bangladesh (2004–2011). P for interaction based on likelihood ratio test of “period” by “exposure to polluting fuels”. Odds ratios (OR) for linear trend (*i.e.* the period effects) for each exposure group (clean or polluting fuel) were adjusted for wealth index, place of residence, mother’s age, mother’s education, mother’s working status, breastfeeding status and household wall material.

The under-five mortality rate decreased from 6.5% in 2004 to 4.4% in 2011, infant mortality rate from 5.5% in 2004 to 4.0% in 2011, and the neonatal mortality rate from 3.6% in 2004 to 3.0% in 2011 (Figure 1). Use of polluting fuels for cooking was associated with a higher risk of neonatal mortality (OR = 1.49, 95% CI = 1.01–2.22, *p* = 0.043) than infant (OR = 1.27, 95% CI = 0.91–1.77, *p* = 0.157) and under-five mortality (OR = 1.14, 95% CI = 0.83–1.55, *p* = 0.422) after adjusting for household wealth, place of residence, mother’s age, mother’s education, mother’s working status, breastfeeding status and wall material of house (Table 1).

Our stratified analysis showed no strong evidence of association between using polluting fuels for cooking and under-five mortality in households in urban compared to rural areas (Table 2). However, there was slightly higher association between use of polluting fuels and neonatal, infant and under-five mortality for those living in urban areas than rural areas (Table 2). Analyses showed a more than four-fold higher risk of mortality in women who never breastfed and who used polluting fuels for cooking (compared to breastfeeding women who used clean fuels), with stronger associations evident for neonatal (OR = 5.39, 95% CI = 2.94–9.92, *p* < 0.001) than infant (OR = 4.47, 95% CI = 2.83–7.06, *p* < 0.001) and under-five mortality (OR = 4.21, 95% CI = 2.77–6.41, *p* < 0.001) (Table 2). The risk of child mortality was also higher for the women who ever breastfed but used polluting fuel for cooking (Table 2).

Analyses combining location of kitchen (outdoors or in the house) and use of cooking fuels for the years 2007–2011 (periods where information on location of kitchen was available), showed a stronger association between households using polluting fuels and an indoor kitchen (compared to households with an outside kitchen and clean fuel use for cooking), with stronger associations for under-five mortality (OR = 1.88, 95% CI = 1.23–2.87, *p* = 0.003) than infant (OR = 1.69, 95% CI = 1.09–2.62, *p* = 0.019) and neonatal mortality (OR = 1.59, 95% CI = 0.94–2.69, *p* = 0.084) (Table 2). There was also an indication of association between use of polluting fuels in an outside kitchen for neonatal, infant and under-five mortality (Table 2). A sensitivity analysis that included kerosene as a “clean fuel” did not change results (only 0.10% of women reported the use of kerosene for cooking) (data not shown).

**Table 1:** HAP from cooking fuels associated with neonatal, infant and under-five mortality in Bangladesh: a pooled analysis for 2004–2011.

	Neonatal				Infant				Under-Five			
Study Factors	*n*/*N* ^^^	*n* (%) ^P^	OR ^a^ 95% CI	*p* Value	*n*/*N* ^^^	*n* (%) ^P^	OR ^a^ 95% CI	*p* Value	*n*/*N* ^^^	*n* (%) ^P^	OR ^a^ 95% CI	*p* Value
**Type of Cooking Fuel**												
Clean fuel ^R^*	41/1843	2.2	1.00		62/1843	3.5	1.00		73/1843	4.2	1.00	
polluting fuel **	532/16,465	3.3	1.49 (1.01–2.22)	0.043	740/16,465	4.6	1.27 (0.91–1.77)	0.157	850/16,465	5.2	1.14 (0.83–1.55)	0.422
**Place of Residence**												
Urban	176/5888	3.0	1.00		250/5888	4.3	1.00		284/5888	5.0	1.00	
Rural	397/12420	3.2	1.01 (0.81–1.25)	0.967	552/12,420	4.5	0.96 (0.80–1.16)	0.690	639/12,420	5.2	0.98 (0.82–1.17)	0.799
**Wealth Index**												
Rich ^R^	95/3512	2.7	1.00		125/3512	3.5	1.00		137/3512	3.9	1.00	
Middle	198/6591	3.1	0.98 (0.72–1.33)	0.904	272/6591	4.2	1.03 (0.79–1.35)	0.822	316/6591	4.8	1.13 (0.87–1.46)	0.367
Poor	280/8205	3.4	1.12 (0.79–1.59)	0.535	405/8205	5.0	1.15 (0.84–1.57)	0.373	470/8205	5.8	1.25 (0.93–1.69)	0.137
**Mother’s Age**												
40–49 ^R^	10/492	1.9	1.00		23/492	4.7	1.00		27/492	5.6	1.00	
<20	115/2669	4.4	3.88 (1.98–7.59)	<0.001	157/2669	6.1	2.65 (1.65–4.24)	<0.001	171/2669	6.6	2.39 (1.54–3.72)	<0.001
20–29	347/11,266	3.2	2.08 (1.09–3.96)	0.027	471/11,266	4.3	1.40 (0.88–2.13)	0.163	542/11,266	4.9	1.34 (0.89–2.02)	0.164
30–39	101/3865	2.6	1.61 (0.83–3.11)	0.161	151/3865	3.9	1.09 (0.69–1.72)	0.717	183/3865	4.8	1.12 (0.73–1.71)	0.602
**Mother’s Education**												
Secondary or higher ^R^	236/7987	3.1	1.00		295/7987	3.8	1.00		335/7987	4.4	1.00	
Primary	181/5673	3.2	1.15 (0.93–1.44)	0.203	248/5673	4.3	1.25 (1.03–1.52)	0.024	284/5673	4.9	1.22 (1.02–1.47)	0.029
No education	156/4648	3.4	1.28 (1.01–1.63)	0.047	259/4648	5.7	1.67 (1.36–2.04)	<0.001	304/4648	6.5	1.64 (1.36–1.99)	<0.001
**Mother’s Working Status**												
Working ^R^	110/3113	3.6	1.00		162/3113	5.2	1.00		186/3113	6.0	1.00	
Not working	463/15,195	3.1	0.87 (0.69–1.08)	0.208	640/15,195	4.3	0.85 (0.71–1.03)	0.100	737/15,195	5.0	0.86 (0.72–1.03)	0.092
**Breastfeeding Status**												
Ever breastfed	260/123,377	2.1	1.00		346/12,377	2.8	1.00		408/12,377	3.3	1.00	
Never breastfed	313/5931	5.5	3.02 (2.52–3.62)	<0.001	456/5931	8.1	3.40 (2.91–3.97)	<0.001	515/5931	9.2	3.23 (2.79–3.73)	<0.001
**Wall Material of House**												
Cement/brick ^R^	248/8470	2.9	1.00		322	3.8	1.00		362/8470	4.2	1.00	
Non-cement/non-brick	279/8528	3.4	0.93 (0.73–1.18)	0.564	420	5.1	1.02 (0.83–1.25)	0.866	494/8528	6.0	1.04 (0.85–1.26)	0.727
**Year of Survey**												
2004 ^R^	199/5852	3.6	1.00		308	5.5	1.00		364/5852	6.5	1.00	
2007	151/4954	3.1	0.87 (0.68–1.11)	0.260	201	4.2	0.76 (0.62–0.94)	0.012	229/4954	4.8	0.74 (0.61–0.91)	0.003
2011	223/7502	2.9	0.84 (0.67–1.07)	0.157	293	3.8	0.77 (0.63–0.94)	0.010	330/7502	4.3	0.73 (0.61–0.89)	0.001

**^R^** Reference category; ^a^ odds ratio adjusted for wealth index, place of residence, mother’s age, mother’s education, mother’s working status, breastfeeding status and wall material of house; * clean fuels: electricity, liquid petroleum gas (LPG), natural gas, biogas; ** Polluting fuels: kerosene, coal/lignite, charcoal, wood, straw/shrubs/grass, agricultural crop and animal dung; ^^^ *n* = no. of mortality cases and *N* = total number of children for neonatal, infant and under-five age-group; ^p^ percentage of mortality cases.

**Table 2:** Risk of mortality by geographical region, breastfeeding status and kitchen location.

		Neonatal			Infant				Under-five			
Study Factors	*n*/*N* ^^^	*n* (%) ^P^	OR ^a^ 95% (CI)	*p* Value	*n*/*N* ^^^	*n* (%) ^P^	OR ^a^ 95% (CI)	*p* Value	*n*/*N* ^^^	*n* (%) ^P^	OR ^a^ 95% (CI)	*p* Value
**Combined Association of Residence and Use of Cooking Fuel**												
Urban residence used clean fuels ^R^*	38/1703	2.1	1.00		57/1703	3.4	1.00		65/1703	3.9	1.00	
Urban residence used polluting fuels **	138/4185	3.5	1.49 (1.03–2.14)	0.033	193/4185	4.8	1.39 (1.03–1.88)	0.033	219/4185	5.6	1.38 (1.03–1.84)	0.028
Rural residence used clean fuels *	3/140	3.0	0.99 (0.29–3.19)	0.958	5/140	4.5	1.09 (0.42–2.78)	0.863	8/140	6.9	1.56 (0.72–3.37)	0.255
Rural residence used polluting fuels **	394/12,280	3.3	1.45 (1.03–2.04)	0.032	547/12,280	4.5	1.35 (1.02–1.79)	0.036	631/12,280	5.2	1.37 (1.05–1.78)	0.021
**Combined Association of Breastfeeding Status and Use of Cooking Fuel**												
Ever breastfed & used clean fuels ^R^*	11/1024	1.2	1.00		20/1024	2.1	1.00		24/1024	2.6	1.00	
Ever breastfed & used polluting fuels**	249/11,353	2.2	2.05 (1.12–3.78)	0.020	326/11353	2.9	1.49 (0.94–2.36)	0.089	384/11,353	3.4	1.46 (0.96–2.22)	0.080
Never breastfed & used clean fuels *	30/819	3.4	3.49 (1.74–7.02)	<0.001	42/819	5.2	2.73 (1.59–4.69)	<0.001	49/819	6.3	2.68 (1.63–4.41)	<0.001
Never breastfed & used polluting fuels**	283/5112	5.8	5.39 (2.94–9.92)	<0.001	414/5112	8.5	4.47 (2.83–7.06)	<0.001	466/5112	9.6	4.21 (2.77–6.41)	<0.001
Combined Association of Kitchen Location and Use of Cooking Fuel***												
Outside kitchen used clean fuels R*	18/835	2.3	1.00		26/835	3.3	1.00		28/835	3.7	1.00	
Outside kitchen used polluting fuels **	342/10,791	3.1	1.46 (0.89–2.38)	0.130	450/10,791	4.1	1.33 (0.89–2.01)	0.167	497/10,791	4.5	1.35 (0.91–2.02	0.134
Inside kitchen used clean fuels *	12/621	1.5	0.89 (0.42–1.87)	0.754	14/621	1.6	0.72 (0.37–1.39)	0.33	20/621	2.7	0.96 (0.53–1.73)	0.895
Inside kitchen used polluting fuels **	82/2383	3.1	1.59 (0.94–2.69)	0.084	126/2383	5.1	1.69 (1.09–2.62)	0.019	151/2383	6.1	1.88 (1.23–2.87)	0.003

**^R^** Reference category; ^a^ odds ratio; * clean fuels: electricity, liquid petroleum gas (LPG), natural gas, biogas; ** Polluting fuels: kerosene, coal/lignite, charcoal, wood, straw/shrubs/grass, agricultural crop and animal dung; ^^^ *n* = number of mortality cases and *N* = total number of children for neonatal, infant and under-five age-group, ^p^ percentage of mortality cases; *** Analyses by these combined factors (location of kitchen and type of cooking fuel) were restricted for the year 2007–2011.

## 4. Discussion

Under-five mortality in Bangladesh has decreased significantly over time, and the relative effect of household use of polluting fuels for cooking on differentials in under-five mortality has not changed substantially. The risk of death was higher in neonatal and infant age groups, and was generally consistent with previous studies in India and Nigeria [29,36]. The risk of death also increased with a proxy measure of the level of exposure to HAP, with higher risk evident in households with indoor kitchens using polluting fuel compared to either indoor or outdoor kitchens using clean fuels. This was because young children less than one year of age were more likely to be indoors or be carried by mother and exposed to higher levels of HAP while cooking [2,10,13]. These findings are biologically plausible as polluted cooking fuels had high range of key pollutants such as fine particles, carbon monoxide (CO) and a number of other chemicals than clean cooking fuels which increased the risk of death among children. The risk of under-five mortality in all age-groups was slightly higher in urban areas compared to rural areas and in households with indoor (compared to outdoor) kitchens. There was also a lower risk of mortality in mothers who had ever breastfed compared to never breastfed which was consistent with its previously reported role in respiratory outcomes [38,40,41] and which appeared to attenuate the effects of polluting fuel use, with a higher risk of under-five mortality among never breastfeeding (compared to ever breastfeeding) mothers using polluting cooking fuels.

This was the first assessment of HAP and under-five mortality in Bangladesh. In this study, we found urban children were at slightly higher risk of death than children from rural areas, despite the fact that most rural households (98%) and 63% of urban households use polluting fuels for cooking. A previous study from Indonesia conducted similar analyses of the association between HAP and under-five mortality for urban and rural areas and identified that neonatal mortality was higher in urban areas than rural areas [30]. HAP related studies in Bangladesh had predominantly focused on non-fatal respiratory infections, with these studies showing impacts of exposure to cooking fuel on child health mainly in urban areas [18,19,20,21,22,23]. One of those studies compared urban and rural areas with HAP and the health of children in households using biomass fuels and identified pollutant levels were higher in urban households [21]. Although cleaner fuel was common in urban areas, in most of the urban slums and peri-urban areas biomass fuel was used along with primarily cleaner fuels as there was always a shortage of cleaner fuels (natural gas/LPG) [19].

Moreover, house and kitchen characteristics were different in urban and rural areas. In urban areas, wall and roof materials in dwellings are more commonly brick and tin whereas in rural areas the wall and roof materials are wood, thatch, mud or bamboo [21]. In many instances, broken sections in the bamboo or wood wall create natural ventilation to reduce the effects of HAP [20]. Dwellings in urban areas are located in close proximity with one another, while in rural areas household are more widely spaced at a distance allowing more air flow and a more rapid dissemination of pollutants [21]. Other factors like a greater number of people in rooms, fewer windows and doors, and the location of kitchen (in the house) may also increase the risk of child deaths in peri-urban areas from exposure to cooking fuels.

Breastfeeding has previously been shown to protect infants against infection and has been reported as a protective factor for reducing risk of respiratory illness among infants [38,40], and thus was a behavior that might attenuate the association between HAP and child mortality. Analyses that combined breastfeeding status also found substantial differences in the association between HAP and under-five mortality between women who did or did not breastfeed. Analyses that incorporated the location of kitchen found a higher risk of neonatal, infant and under-five mortality when mothers reported an inside kitchen and used polluting fuels cooking. Children from poorer socio-economic conditions (such as those from poorer households, and mothers with no education and poor household materials) and mother’s age (such as younger mother) were also found to be at greater risk of death from exposure in all age groups, which was also consistent with previous similar studies [27,28,30,31,32,33,35,36].

There are a number of methodological considerations to be taken into account when interpreting these findings. This was a cross-sectional study design based on secondary data, with a number of potential sources of bias including selection, misclassification and recall bias. Firstly, the classification of cooking fuel may be a source of misclassification bias, as some households use a combination of polluting and clean fuels. Moreover, the DHS survey only collected information of primary fuel use, and there was no data for secondary fuel use. One recent study on Bangladesh indicated that dwellings reporting gas as their primary fuel frequently shift to cooking with biomass fuels during shortages of gas supply which may cause higher concentrations of HAP [19], and attenuate associations between HAP and child mortality. In addition, this study did not account for past exposure to HAP or recent changes in cooking methods because of its cross-sectional design.

Secondly, information on birth and death of children was self-reported by mothers, which may be a source of recall bias. The present study constrained analyses to those children born within a five year period prior to the survey date in order to minimize the likelihood of recall bias (and maximize the study sample size). Thirdly, this study used cross-sectional data for analysis, and it is difficult to clearly define temporal relationships between the exposure and outcome when collected at the same point of time. Another limitation of this current analysis was its relatively small sample size and lack of statistical power. Because of the small number of cases, measures of association had wide confidence intervals when analyses were stratified by geographical location (particularly analyses for rural areas), breastfeeding status and kitchen location.

Furthermore, we considered all-cause mortality for our analysis of association between HAP and under-five mortality. Cause of death information was not available in the BDHS dataset used for this study. Verbal autopsy questionnaires are employed for identifying cause in Bangladesh, however this information is of variable quality and were not collected for all years (not available in BDHS 2007). Not only pneumonia and acute respiratory infections but also other factors, such as preterm birth complications, low birth weight, nutritional conditions and diarrhea also affects mortality among under-five children. On the basis of 2011 BDHS report, pneumonia remained the largest single cause of under-five deaths in Bangladesh, accounting for one-fifth of all deaths [14]. The 2010 Global Burden of Disease (GBD) study also indicated that acute lower respiratory infections was the leading cause of death in infancy period and second leading cause of death (after drowning) in under-five children in Bangladesh [42]. HAP from cooking fuel is a primary cause for respiratory infections among under-five children and thus can be associated with the deaths caused by those illnesses. However, including all-cause mortality will also include mortality outcomes not associated with HAP, which is likely to be a source of ascertainment bias in the outcome, and lead to an underestimation of the association between HAP and the cause-specific outcomes noted above.

This study also did not measure actual levels of exposure to emission from cooking smoke. Proxy environmental and behavioral measures were defined to examine the effects of level of exposure. Location of kitchen has previously been investigated to be an important factor in studies of developing countries associated with HAP and under-five mortality [28,32,33], which was also consistent with observed associations between HAP and under-five mortality in the present study (restricted to the two surveys—2007 and 2011—Where this information was available). Several studies in Bangladesh have noted a significant association between location of kitchen and HAP [19,21,22,23,37,43].

Despite these methodological concerns, this present study used large-scale nationally representative DHS data with a very high response rate of 98.3%, and was the first study to pool datasets over a long period (2004–2011), a period in Bangladesh characterized by rapid socio-economic development. No previous studies have investigated trends and differentials in the association between HAP and under-five mortality in Bangladesh over this long period, or assessed the role of environmental and behavioral factors that may be points of intervention and health promotion at the national level in the Bangladeshi context.

The magnitude of the association between HAP and under-five mortality remained consistent over time in all age-groups. However, increased exposure to cooking fuel can raise the risk of respiratory illness and child deaths in developing countries like Bangladesh. Studies have found that there is little use of cleaner fuels in rural areas [43] and 50% of urban and peri-urban households still rely on polluting fuels for domestic energy [14]. In addition, with only 14% of the total population having access to electricity, Bangladesh has the lowest per capita energy consumption in South Asia where the major primary energy source is biomass/wood (57%), with the remainder being supplied from natural gas (29%) [44,45]. Despite the potentially small relative risk of under-five mortality associated with HAP, it remains a common exposure in the population and therefore the population attributable risk—of this preventable risk factor—is a public health priority for Bangladesh.

Awareness-raising of the health risk related with HAP and the use of polluting fuel is needed in rural and low income urban areas. Radio and television have been identified as useful media to raise awareness and to reach poorer sub-groups of the population [45,46]. Many countries like Brazil, Bolivia, and Ecuador have decreased exposure to pollution from cooking fuel by promoting liquid fuel supported by government policy [31,47]. Switching to clean fuel is advisable, however, it is an expensive option for many poor families in Bangladesh [43]. Behavioral change interventions would also have potential in Bangladesh to reduce child exposure from HAP [48]. Studies from Bangladesh suggested that improvements in natural household ventilation, particularly windows and improved stoves (for example, new biofuel stove design) might lead to a reduction in HAP [23,37,43]. In the Bangladesh context, behavioral interventions are likely to play an important role in decreasing childhood deaths by promoting the use of improved cooking stoves, increased natural household ventilation, and to not carry babies while cooking.

## 5. Conclusions

Bangladesh is a country where overall under-five mortality rates have decreased substantially over the study period (2004–2007). While HAP was associated with a modest increase in risk of mortality in children under five, the ubiquitous use of solid cooking fuel in Bangladesh and associated population attributable risk, confirms HAP as an important public health problem. Cooking fuel is a modifiable risk factor that can be changed by improvements in house design, health system policies, infrastructure, behavioral intervention and economic development.

## Supplementary Files

Supplementary File 1
