# Supplementary material for: Household Air Pollution and Under-Five Mortality in Bangladesh (2004–2011)
Source: Int J Environ Res Public Health. 2015 Oct 15;12(10):12847–62. doi: 10.3390/ijerph121012847 (PMC4627003; doi:10.3390/ijerph121012847)
Supplement: Supplementary File 1 [file ijerph-12-12847-s001.pdf]

Supplementary Information

Household Air Pollution and Under-Five Mortality in Bangladesh (2004–2011)

Table S1. Total number and proportion of women by study characteristics for each year of the survey.

	2004		2007		2011	
Study Factors	N	N (%)	N	N (%)	N	N (%)
Type Of Cooking Fuel						
Clean Fuel	453	7.74	481	9.71	909	12.12
Polluting Fuel	5399	92.26	4473	90.29	6593	87.88
Wealth Index						
Rich	827	14.13	918	18.53	1767	23.55
Middle	1878	32.09	1731	34.94	2982	39.75
Poor	3147	53.78	2305	46.53	2753	36.7
Mother's Age						
40–49	167	2.86	118	2.38	207	2.76
<20	971	16.61	768	15.5	930	12.41
20–29	3445	58.94	3032	61.2	4789	63.91
30–39	1262	21.59	1036	20.91	1567	20.91
Mother's Education						
Secondary Or Higher	2022	34.55	2213	44.67	3752	50.01
Primary	1796	30.69	1553	31.35	2324	30.98
No Education	2034	34.76	1188	23.98	1426	19.01
Mother's Working Status						
Working	1062	18.15	1309	26.42	742	9.89
Not Working	4790	81.85	3645	73.58	6760	90.11
Sex Of Child						
Female	2873	49.09	2461	49.68	3651	48.67
Male	2979	50.91	2493	50.32	3851	51.33
Wall Material Of House *						
Cement/Brick	1072	18.32	2668	53.86	4730	63.05
Non-Cement/Non-Brick	4770	81.51	1773	35.79	1985	26.46
Breastfeeding Status						
Ever Breastfed	4141	70.76	3376	68.15	4860	64.78
Never Breastfed	1711	29.24	1578	31.85	2642	35.22
Place Of Residence						
Urban	1802	30.79	1755	35.43	2331	31.07
Rural	4050	69.21	3199	64.57	5171	68.93
Location Of Kitchen **						
Outside Of House	.	.	4542	75.05	7084	82.44
Inside Of House	.	.	1499	24.77	1505	17.51

* Percentage did not add up to 100% because of missing values; ** No observation was available before year 2007 and percentage did not add up to 100% because of missing values.
